# Mechanism of [CO_2_] Enrichment Alleviated Drought Stress in the Roots of Cucumber Seedlings Revealed via Proteomic and Biochemical Analysis

**DOI:** 10.3390/ijms232314911

**Published:** 2022-11-28

**Authors:** Yiman Li, Wendong Zhang, Dalong Zhang, Yinjian Zheng, Yaliang Xu, Binbin Liu, Qingming Li

**Affiliations:** 1 College of Horticulture Science and Engineering, Shandong Agricultural University, Tai’an 271018, China; 2 College of Water Resource and Civil Engineering, China Agricultural University, Beijing 100083, China; 3 State Key Laboratory of Crop Biology, Tai’an 271018, China; 4 Institute of Urban Agriculture, Chinese Academy of Agricultural Sciences, Chengdu 610299, China

**Keywords:** cucumber roots, CO_2_ enrichment, drought stress, TMT-based quantitative proteomic, carbohydrate synthesis, amino acid metabolism

## Abstract

Cucumber is one of the most widely cultivated greenhouse vegetables, and its quality and yield are threatened by drought stress. Studies have shown that carbon dioxide concentration ([CO_2_]) enrichment can alleviate drought stress in cucumber seedlings; however the mechanism of this [CO_2_] enrichment effect on root drought stress is not clear. In this study, the effects of different drought stresses (simulated with 0, 5% and 10% PEG 6000, i.e., no, moderate, and severe drought stress) and [CO_2_] (400 μmol·mol^−1^ and 800 ± 40 μmol·mol^−1^) on the cucumber seedling root proteome were analyzed using the tandem mass tag (TMT) quantitative proteomics method. The results showed that after [CO_2_] enrichment, 346 differentially accumulating proteins (DAPs) were found only under moderate drought stress, 27 DAPs only under severe drought stress, and 34 DAPs under both moderate and severe drought stress. [CO_2_] enrichment promoted energy metabolism, amino acid metabolism, and secondary metabolism, induced the expression of proteins related to root cell wall and cytoskeleton metabolism, effectively maintained the balance of protein processing and degradation, and enhanced the cell wall regulation ability. However, the extent to which [CO_2_] enrichment alleviated drought stress in cucumber seedling roots was limited under severe drought stress, which may be due to excessive damage to the seedlings.

## 1. Introduction

Drought is a major environmental factor affecting crop growth and yield worldwide [1], and the arid and semi-arid areas threatened by irrigation water shortages account for about 50% of China’s land area [2]. Meanwhile, atmospheric carbon dioxide concentration ([CO_2_]) has exceeded 415 μmol·mol^−1^ (https://www.CO2.earth/ accessed on 30 January 2022) and are predicted to rise to 700–800 μmol·mol^−1^ by the end of the century (IPCC 2013).

Under drought stress, [CO_2_] enrichment can improve plant growth rate and yield by increasing photosynthetic rate, reducing stomatal conductance and respiration, and increasing water use efficiency, thereby alleviating the degree of plant stress and producing a significant “CO_2_ fertilization effect” [3]. Previous studies on plant responses to [CO_2_] enrichment have focused on the growth and development, physiology and metabolism, and yield and quality of above-ground organs of crops [4]. As one of the important organs of terrestrial plants, changes in environmental conditions inevitably cause changes in the root system, which in turn affect the function of crop uptake and transport of water and nutrients. One study showed that [CO_2_] enrichment promoted total fine root length (+44%) and observed root number (+39%) in mixed heathland and grassland [5]. Another study showed that under drought condition, increasing [CO_2_] to 550 μmol·mol^−1^ significantly promoted carbohydrate synthesis in maize leaves, which was then transported to the root system to stimulate root growth and alter root physiological activity [6]. Cucumber (*Cucumis sativus* L.) is one of the most important vegetable crops grown in greenhouses around the world, and its shallow-rooted biology dictates high water requirements. Our previous results showed that under [CO_2_] enrichment conditions, the root phenotype of cucumber seedlings was significantly altered, root biomass, total length, total surface area, and total volume were increased, endogenous phytohormone contents and antioxidant capacity were regulated, and hydraulic conductivity was improved, ultimately mitigating the negative effects of drought stress on the cucumber seedling roots [7,8]. These studies can provide valuable references for understanding [CO_2_] enrichment to improve root drought resistance.

In recent years, comparative proteomics have been used to study the mechanisms by which the root system responds to drought stress and/or [CO_2_] enrichment. For example, plant roots under drought stress are rich in proteins related to carbon and nitrogen metabolism, such as malate dehydrogenase (MDH), α-mannosidase, UDP-sugar pyrophosphorylase, and UDP-glucose-6-phosphate dehydrogenase, suggesting that the root system releases energy to enhance intercellular activity under stress [9,10]. Antioxidant-related proteins such as dehydroascorbate reductase, quinone reductase, and glutathione-S-transferase have been detected in roots under drought stress [11,12], as have molecular chaperones such as HSP70, HSP60, GroEL, and other heat shock proteins that prevent the accumulation and folding of inactive proteins to protect the normal growth and development of root cells under stress [13,14,15]. Proteins related to stress defense as well as those related to protein folding, modification, and degradation have been found to respond to H_2_O_2_-ABA-induced adventitious root development in cucumber under drought stress [16]. In addition, another study has shown that nitrogen metabolism (glutamine synthetase), energy metabolism (glyceraldehyde-3-phosphate dehydrogenase), antioxidant metabolism (ascorbate peroxidase, superoxide dismutase and catalase), and chaperone protection (HSP81-1) proteins respond to drought stress in creeping bentgrass roots under [CO_2_] enrichment conditions [17].

However, few studies have been reported on the proteome of cucumber root systems under [CO_2_] enrichment and drought stress. Therefore, this study aims to analyze the differentially accumulating proteins (DAPs) associated with drought resistance in cucumber seedling roots under [CO_2_] enrichment using the tandem mass tag (TMT) technique. In addition, we analyze the changes in compounds associated with the carbon and nitrogen metabolisms. We expect to provide new insights into [CO_2_] enrichment regulatory mechanisms in order to improve the drought resistance of cucumber seedlings and provide a stronger theoretical basis for CO_2_ fertilization in greenhouse cultivation.

## 2. Results

### 2.1. Overview of Quantitative Proteomic Responses to [CO_2_] Enrichment and Drought Stress

**Figure 1:**
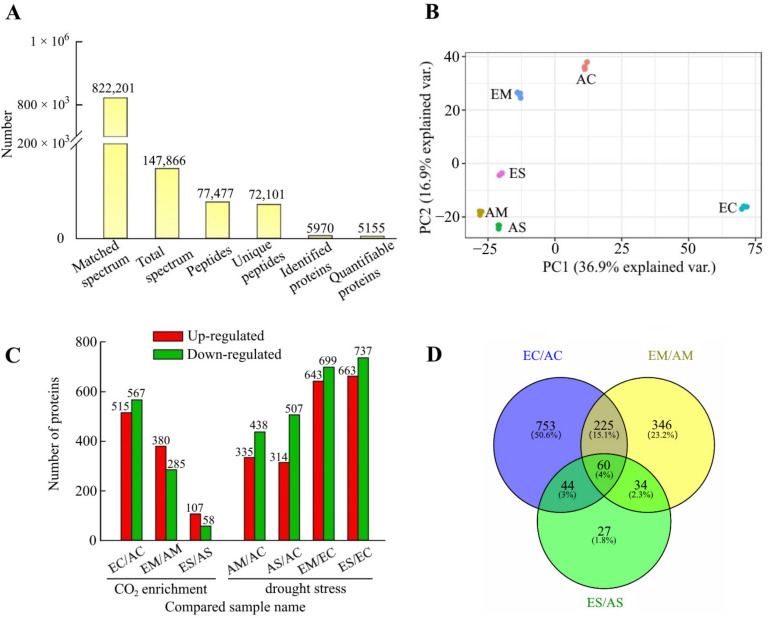
Quantitative proteome analysis of MS data and identification of differentially accumulating proteins. (**A**) The basic statistics of the MS results. (**B**) Principal component analysis of all samples using quantified proteins. (**C**) Histogram of the numerical distribution of differentially accumulating proteins in different comparison groups. (**D**) Venn diagram analysis of differentially accumulating proteins under [CO_2_] enrichment. Matched spectrum, number of spectrum matched with alignment protein; Total spectrum, number of spectrum produced by mass spectrometer; Peptides, number of peptides which spectrum hit; Unique peptides, number of identified peptides that only come from this protein group; Identified proteins, number of proteins detected by spectrum search analysis; Quantifiable proteins, number of proteins quantifiable; AC, atmospheric [CO_2_] + control condition; EC, [CO_2_] enrichment + control condition; AM, atmospheric [CO_2_] + moderate drought stress; EM, [CO_2_] enrichment + moderate drought stress; AS, atmospheric [CO_2_] + severe drought stress; ES, [CO_2_] enrichment + severe drought stress. The same definitions hold below.

In this experiment, the whole proteome of cucumber seedling roots under six treatments was quantified. In total, 77,477 peptides were inferred, of which 72,101 unique peptides had similarities to proteins; after removing duplicates, 5970 proteins were identified, of which 5155 contained quantifiable information (Figure 1A, Table S1). The quantitative repeatability of the proteins was assessed by principal component analysis (PCA), and the degree of aggregation between replicate samples showed the high repeatability of our experiments (Figure 1B). For a more comprehensive analysis of the effect of [CO_2_] enrichment on cucumber seedling roots under drought stress using fold change >1.2 and the *p*-value < 0.05 as screening criteria (Table S2), quantifiable proteins under different [CO_2_] comparison groups (EC/AC, EM/AM, ES/AS) and different drought stress comparison groups (AM/AC, AS/AC, EM/EC, ES/EC) were selected as DAPs (Figure 1C, Table S3). Venn diagrams were established to visualize the relationship of DAPs with [CO_2_] enrichment and different levels of drought stress. The results showed that after [CO_2_] enrichment, 60 DAPs were found under all drought conditions, 34 DAPs under moderate and severe drought stress, 346 DAPs under moderate drought stress, and 27 DAPs under severe drought stress (Figure 1D, Table S4). Venn diagrams of DAPs under different drought stress are shown in Figure S1A and Table S4; these reflect the commonalities and differences in biochemical changes in cucumber roots under [CO_2_] enrichment and drought stress.

### 2.2. Hierarchical Clustering and Functional Classification Analysis of DAP Response to [CO_2_] Enrichment and Drought Stress

**Figure 2:**
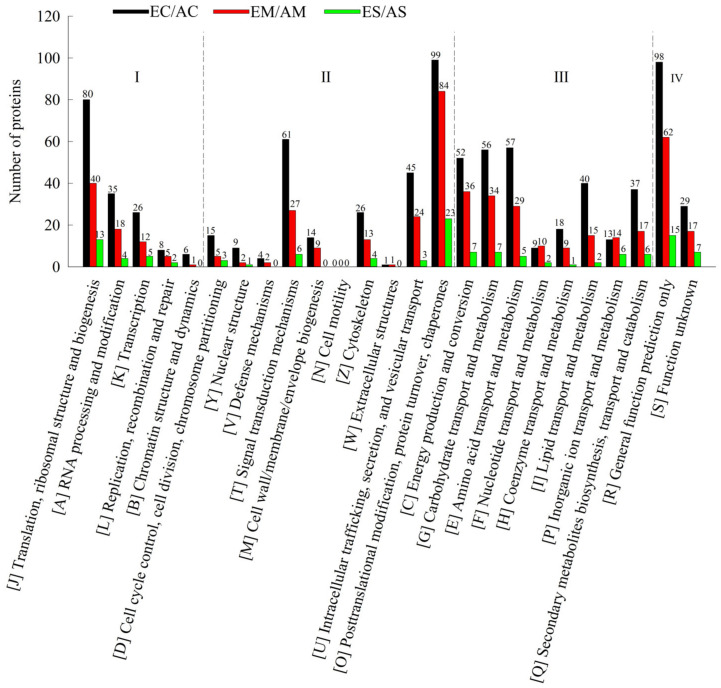
KOG functional classification chart of differential proteins under [CO_2_] enrichment: (I) information storage and processing, (II) cellular processes and signals, (III) metabolism, and (IV) other unknown functions.

In order to obtain a comprehensive understanding of the proteins identified in the data, we annotated the functions and characteristics of these proteins in terms of GO, protein domain, KEGG pathway, KOG functional classification, and subcellular structure localization (Table S5). After functional classification analysis of these DAPs, we found that these DAPs were mainly focused on four aspects: information storage and processing (I), cell process and signaling (II), metabolism (III), and other unknown functions (IV) (Figure 2 and Figure S1B, Table S6).

**Figure 3:**
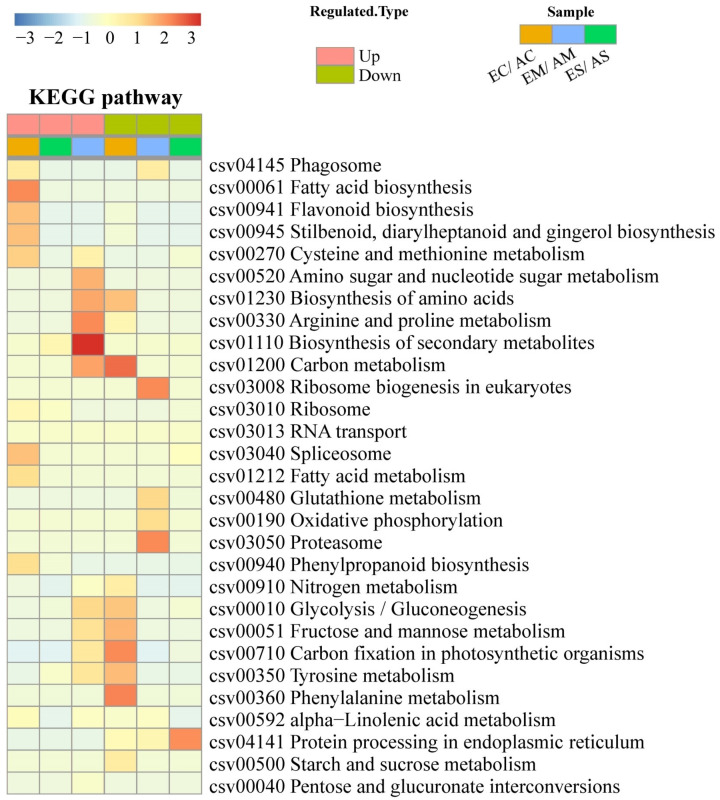
Cluster analysis of the enrichment patterns of KEGG pathways of differential accumulating proteins under [CO_2_] enrichment. The color blocks corresponding to the functional description of the differentially expressed proteins in different groups indicate the degree of enrichment; red represents strong enrichment and blue represents weak enrichment.

Then, the DAPs of each comparison groups were subjected to enrichment analysis and cluster analysis at the level of the KEGG pathways (Table S7). The results showed that after [CO_2_] enrichment, eight metabolic pathways (glycolysis/gluconeogenesis (EMP), fructose and mannose metabolism, arginine and proline metabolism, tyrosine metabolism, amino sugar metabolism and nucleotide sugar metabolism, secondary metabolite biosynthesis, amino acid biosynthesis, and carbon metabolism) were significantly upregulated under moderate drought stress, while only the ribosomal pathway was significantly upregulated under severe drought stress (Figure 3). The changes of metabolic pathways under different drought stress are shown in Figure S2.

**Figure 4:**
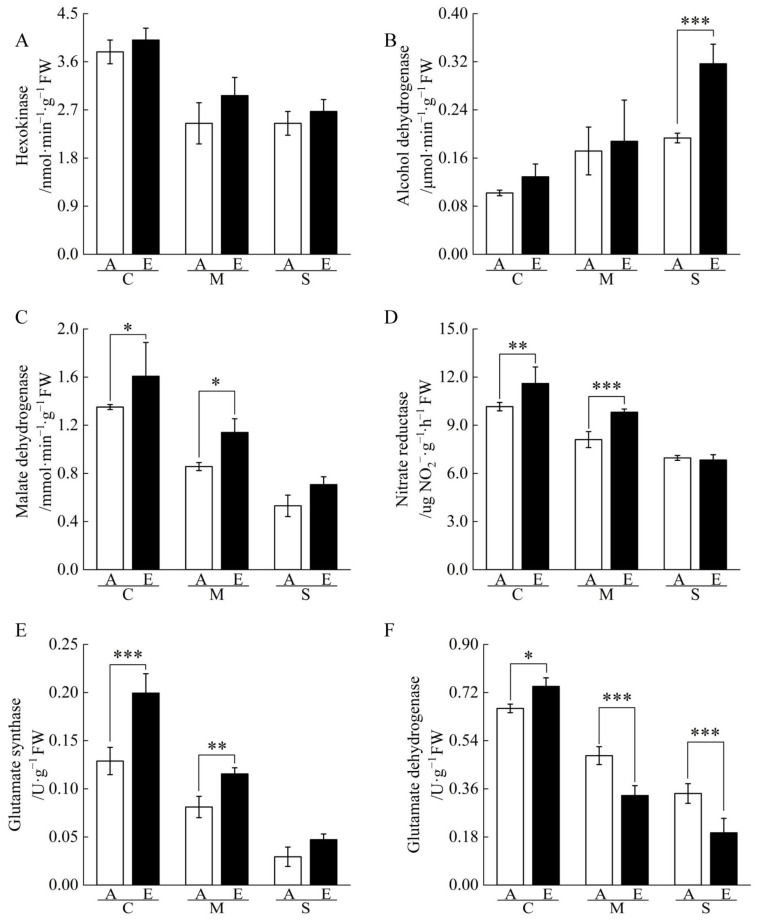
Effects of [CO_2_] enrichment on the activities of related enzymes in roots of cucumber seedlings under drought stress. (**A**) Hexokinase, (**B**) Alcohol dehydrogenase, (**C**) Malate dehydrogenase, (**D**) Nitrate reductase, (**E**) Glutamate synthase, (**F**) Glutamate dehydrogenase. All results are expressed as the mean ± standard deviation (SD) of three repeated values; *, difference is significant at the 0.05 level; **, difference is significant at the 0.01 level; ***, difference is significant at the 0.001 level.

In order to validate the proteomic results, we measured the activity of enzymes in the EMP pathway and in the carbon and nitrogen metabolisms. The results showed that the activities of hexokinase (HK), MDH, nitrate reductase (NR), glutamate synthase (GOGAT), and glutamate dehydrogenase (GDH) decreased under the same [CO_2_] conditions, while the activity of alcohol dehydrogenase (ADH) increased in cucumber seedling roots under drought stress. Under [CO_2_] enrichment conditions, the activities of MDH and NR increased significantly under moderate drought stress, the activity of ADH increased significantly under severe drought stress, and the activity of GDH decreased significantly under moderate and severe drought stress. These results are consistent with those of the proteomic analysis (Figure 4, Table S8).

### 2.3. Non-Structural Carbohydrate Contents of Cucumber Seedling Roots under Drought Stress Changed by [CO_2_] Enrichment

**Figure 5:**
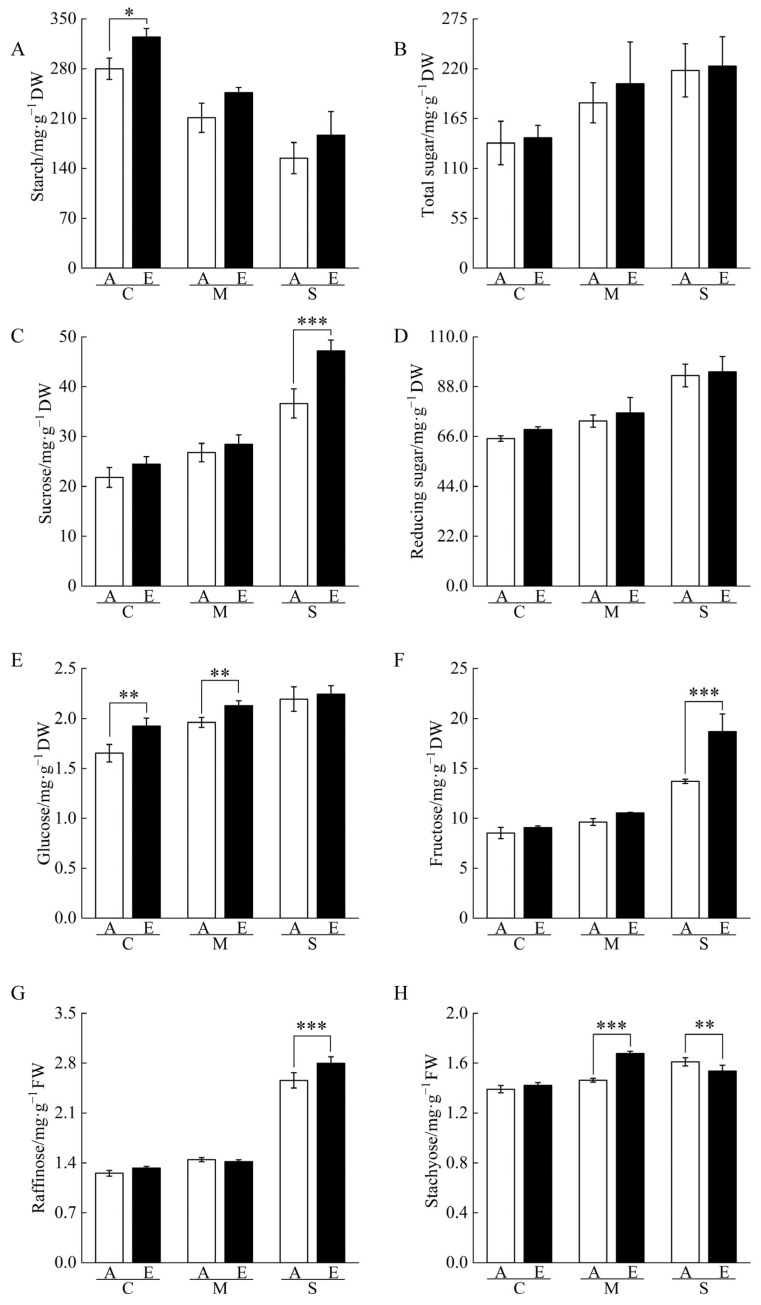
Effects of [CO_2_] enrichment on the contents of non-structural carbohydrates in roots of cucumber seedlings under drought stress. (**A**) Starch, (**B**) Total sugar, (**C**) Sucrose, (**D**) Reducing sugar, (**E**) Glucose, (**F**) Fructose, (**G**) Raffinose, (**H**) Stachyose. All results are expressed as the mean ± standard deviation (SD) of three repeated values; *, difference is significant at the 0.05 level; **, difference is significant at the 0.01 level; ***, difference is significant at the 0.001 level.

Proteomic analysis showed that [CO_2_] enrichment regulated the sugar metabolism pathway in drought-stressed cucumber roots, suggesting that [CO_2_] enrichment may alleviate drought stress by increasing the content of osmoregulators such as sugars. To test this inference, the contents of non-structural carbohydrates in cucumber seedling roots under [CO_2_] enrichment and drought stress were measured (Figure 5, Table S9). Under the same [CO_2_] conditions, the contents of total sugars, sucrose, reducing sugars, glucose, fructose, raffinose, and stachyose in cucumber seedling roots increased with the degree of drought stress, while the starch content decreased with the degree of drought stress. [CO_2_] enrichment significantly enhanced starch and glucose contents under control, glucose and stachyose contents under moderate drought stress, and sucrose, fructose, raffinose, and stachyose contents under severe drought stress compared with atmospheric [CO_2_].

### 2.4. Nitrogen and Organic Acid Contents of Cucumber Seedling Roots under Drought Stress Regulated by [CO_2_] Enrichment

**Figure 6:**
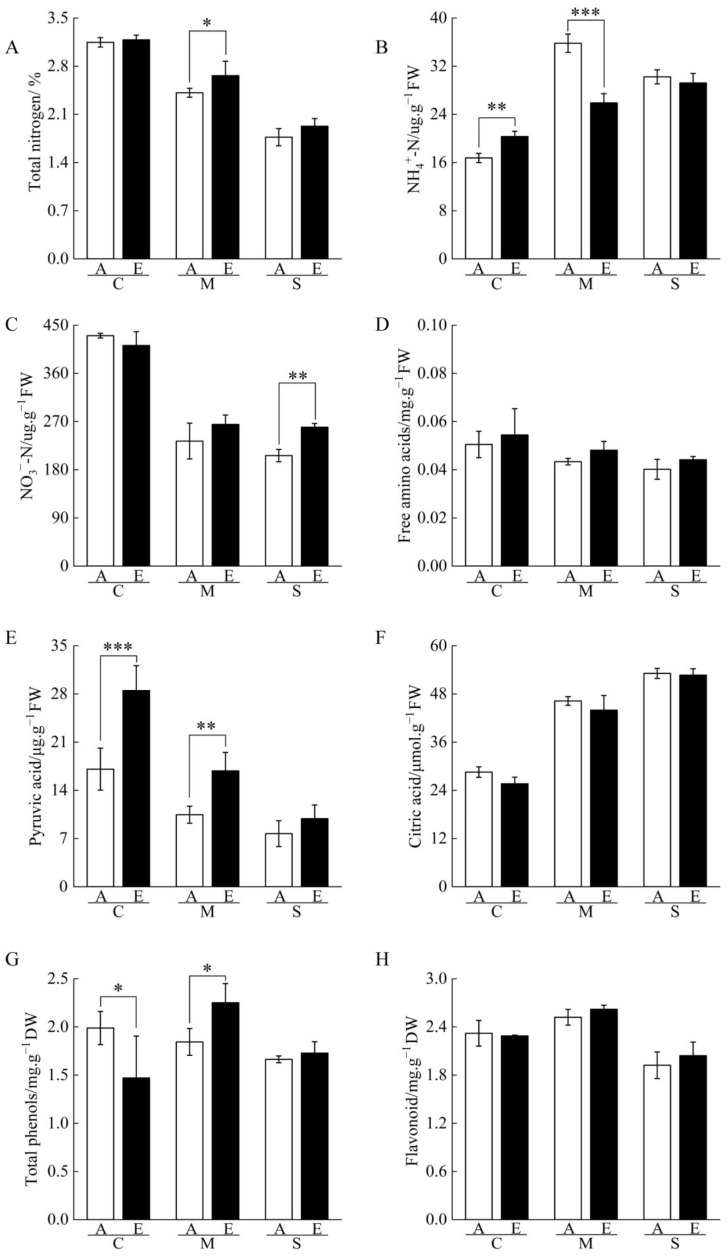
Effects of [CO_2_] enrichment on the contents of metabolism-related compounds in roots of cucumber seedlings under drought stress. (**A**) Total nitrogen, (**B**) NH_4_^+^-N, (**C**) NO_3_^−^-N, (**D**) Free amino acid, (**E**) Pyruvic acid, (**F**) Citric acid, (**G**) Total phenols, (**H**) Flavonoid. All results are expressed as the mean ± standard deviation (SD) of three repeated values; *, difference is significant at the 0.05 level; **, difference is significant at the 0.01 level; ***, difference is significant at the 0.001 level.

Proteomic analysis showed that [CO_2_] enrichment altered the amino acid metabolism and EMP pathway in cucumber seedling roots under drought stress. The relevant compound contents were determined, and it was found that total nitrogen content significantly decreased with the degree of drought stress, while citric acid content significantly increased with the degree of drought stress under the same [CO_2_] conditions. [CO_2_] enrichment significantly increased pyruvic acid content under both control and moderate drought stress, increased total nitrogen content while decreasing NH_4_^+^-N content under moderate drought stress, and increased NO_3_^−^-N content under severe drought stress compared to atmospheric [CO_2_]. In addition, the EM treatment had the highest total phenol and flavonoid contents, and there was no significant difference in free amino acid content between treatments (Figure 6, Table S10).

### 2.5. Correlation Analysis

**Figure 7:**
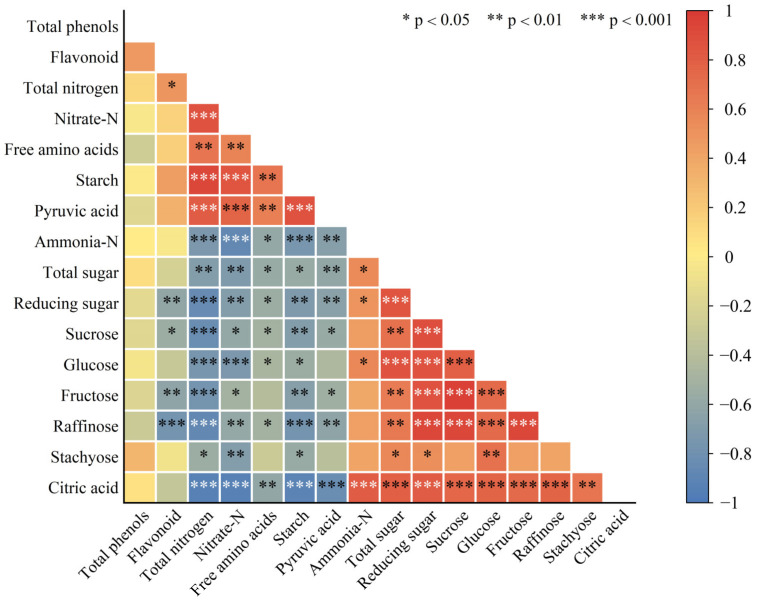
Correlation analysis between biochemical indicators; red indicates positive correlation, blue indicates negative correlation.

Correlation analysis of cucumber seedlings root biochemical indexes of different treatments was performed (Figure 7). The results showed that flavonoid content was significantly and positively correlated with total nitrogen content, while it was significantly and negatively correlated with reducing sugar, sucrose, fructose, and raffinose content. NH_4_^+^-N content was significantly and negatively correlated with total nitrogen, NO_3_^−^-N, and free amino acid content. Starch content was significantly and negatively correlated with total sugar, reducing sugar, sucrose, glucose, fructose, raffinose, and stachyose content. Total nitrogen and total sugar content were significantly and negatively correlated, as were pyruvic acid and citric acid content, while the total content of phenols was not correlated with any of the other indicators.

## 3. Discussion

### 3.1. [CO_2_] Enrichment Improves Carbohydrates Content and Energy Metabolism

In this experiment, we found that many DAPs with reduced abundance in cucumber seedling roots under moderate and severe drought stress were involved in the EMP pathway (Table S7). Previous studies have shown that nearly 20% of the responsive proteins in plant cells under drought stress are related to carbohydrate and energy metabolism [18]. [CO_2_] enrichment increased starch and sucrose contents under drought stress (Figure 5) while increasing the abundance of sugar transport protein (STP) along with twelve key enzymes in the EMP pathway under moderate drought stress (Table S7). Photosynthetic products synthesized in leaves and transported to roots can be converted into non-structural carbohydrates, mainly in the form of soluble sugars and starch, where soluble sugars can be used directly for growth and respiration while starch is used for energy storage [19]. Although drought inhibited CO_2_ fixation and transport in leaves, the relative amount of photosynthetic products allocated to roots increased due to increased expression of STP under [CO_2_] enrichment, which promotes sugar transport from source to sink, reduces cellular osmotic potential, and provides sufficient substrates for the EMP pathway, enhancing the root resistance of cucumber seedling roots to moderate drought stress [20,21,22,23]. Zhou et al. [24] showed that [CO_2_] enrichment can improve the transport of sugars from leaves to stems and promote the survival of the single bud stem of grape. Studies by Calvo et al. [25] on barley and Li et al. [26] on cucumber showed that plants can produce more sugars under [CO_2_] enrichment to increase root biomass as well as the organic acid and amino acid contents.

Among the DAPs associated with the EMP pathway, FK was able to catalyze the transfer of phosphate groups from ATP to fructose and improve the synthesis of cell wall polysaccharides, which repairs the energy deficiency and osmotic damage in plants after drought stress [27]. Pyrophosphate-fructose 6-phosphate 1-phosphotransferase (PFP) catalyzes the ATP-independent transformation between fructose 6-phosphate (F6P) and fructose 1,6-bisphosphate (FBP) to maintain ATP homeostasis under stress [28]. Fructose-bisphosphate aldolase (ALDO) and glyceraldehyde-3-phosphate dehydrogenase (GAPDH) were positively correlated with drought resistance [29,30]. Notably, overexpression of GAPDH-related regulatory genes in rice can control the excessive accumulation of H_2_O_2_ and reduce oxidative stress in cells [31]. Pyruvate decarboxylase (PDC) and ADH catalyze the conversion of pyruvic acid to acetaldehyde, ensuring the continuation of EMP and consuming the NADH produced during EMP to protect cells from acidification [32]; ADH abundance is upregulated under severe drought stress, which was consistent with our enzyme activity determination results (Figure 4).

This study identified that [CO_2_] enrichment increased the abundance of the rate-limiting enzyme MDH in the tricarboxylic acid cycle (TCA cycle) (Table S7, Figure 4); Li et al. [33] have previously shown that [CO_2_] enrichment increases the activity of MDH in cucumber leaves under salt stress. Under moderate drought stress, [CO_2_] enrichment increased the abundance of ribose-5-phosphate isomerase 3, transketolase, and fructose-1,6-bisphosphatase (FBPase) in the pentose phosphate pathway (PPP), while FBPase remained increased under severe drought stress (Table S7), perhaps leading to the inference that [CO_2_] enrichment can enhance the stability of the EMP pathway and TCA cycle under drought stress by promoting the abundance of related proteins in the PPP [34]. In addition, [CO_2_] enrichment increased NADH dehydrogenase abundance under moderate drought stress (Table S7); this change suggests that [CO_2_] enrichment can enhance cellular energy production by increasing the protein abundance associated with the electron transport chain, thereby decreasing drought stress damage by maintaining the normal metabolic activity of the root system as much as possible [35,36].

### 3.2. [CO_2_] Enrichment Improves Amino Acid Metabolism and N Remobilization

In our study, NO_3_^−^ content decreased, NH_4_^+^ content increased, and NR and GOGAT activities decreased under drought stress. This is consistent with Li et al. [37], who studied the metabolic processes in cucumber leaves under salt stress and suggested that the decrease in NR activity was induced by the decrease in NO_3_^−^ content. The reduced activity of NR and GOGAT may then lead to a decreased rate of assimilation of NH_4_^+^ into amino acids, intracellular accumulation and toxicity [38]. We found that [CO_2_] enrichment increased the abundance of NR and GOGAT while decreasing the abundance of GDH under moderate drought stress. The increase in NR abundance may be due to [CO_2_] enrichment alleviating the inhibitory effect of drought stress on root vigor, increasing total biomass in the underground [7,8], and favoring root uptake of inorganic nitrogen, which in turn induce an increase in NR abundance [37]. The glutamine synthetase GS-GOGAT cycle is the main pathway for NH_4_^+^ assimilation in higher plants, and GDH plays a complementary role in the GS-GOGAT cycle [39]. Therefore, in our experiment, the decrease in GDH abundance and increase in GOGAT abundance indicates that cucumber roots under [CO_2_] enrichment mainly eliminated NH_4_^+^ produced under moderate drought stress through GOGAT [40]. [CO_2_] enrichment increased the abundance of adenylate kinase (ADK), linoleate 13S-lipoxygenase, and other key enzymes for amino acid biosynthesis under moderate drought stress (Table S7); these are involved in the synthesis of eleven common amino acids, such as glutamate, methionine, and proline. ADK is a phosphotransferase that catalyzes the interconversion of various adenosine phosphates and plays an important role in cellular energy homeostasis [41], while linoleate 13S-lipoxygenase oxidizes and alpha-linolenate is involved in jasmonic acid synthesis [42]. In short, [CO_2_] enrichment promotes amino acid metabolism and biosynthesis of certain amino acid derivatives under moderate drought stress, which is consistent with the findings of Cui et al. [43] with respect to cucumber leaves.

The S-adenosyl-L-methionine (SAM) cycle provides precursors for ethylene and polyamines, supplies methyl groups for many biomolecules, and plays an important role in enhancing plant drought tolerance [44,45]. After [CO_2_] enrichment, the abundance of 1-aminocyclopropane-1-carboxylate (ACC) synthase (ACS) and ACC oxidase (ACO) in the SAM cycle increased under moderate drought stress (Table S7). Studies have shown that SAM synthesizes ACC through ACS and then ethylene through ACO, and the increase of ACC and ethylene contents can enhance drought resistance [46,47]. These results imply that [CO_2_] enrichment can increase ethylene content in cucumber roots under drought stress; however, in this study, only ACO abundance increased with increasing stress level in the SAM cycle, which may be due to excessive damage to the cucumber roots under severe drought stress resulting in a limited alleviating effect of [CO_2_] enrichment. In our results, the abundance of ubiquitin-conjugating enzyme E2 (UBE2) was upregulated under moderate and severe drought stress after [CO_2_] enrichment. Overexpression of UBE2 in soybean, peanut, and arabidopsis has been found to improve drought tolerance [48,49], suggesting that [CO_2_] enrichment can promote remobilization of amino acids from inactivated proteins to alleviate drought stress in cucumber roots.

### 3.3. [CO_2_] Enrichment Reduces Drought-Induced Damage in Roots

[CO_2_] enrichment can help plants to resist oxidative stress damage by regulating the synthesis of secondary metabolites [50,51]. Kiba et al. [52] reported that [CO_2_] enrichment induced the expression of the anadenosine phosphate–isopentenyltransferase (AtIPT3) and cytochrome P450 monooxygenase (CYP735A2) genes by increasing the sugar content in the root system, leading to an increase in cytokinin content in the root system. Our previous study showed that [CO_2_] enrichment prevented the decreasing trend of cytokinin content under drought stress [8]. In this experiment, after [CO_2_] enrichment, the abundance of 3-hydroxy-3-methylglutaryl-CoA synthase (HMGS) in the mevalonate (MVA) pathway and 1-deoxy-D-xylulose 5-phosphate reductoisomerase (DXR) and 4-hydroxy-3-methylbut-2-enyldiphosphate synthase (HDS) in the 2-C-methyl-D-erythritol 4-phosphate (MEP) pathway were upregulated under moderate drought stress (Table S7). Dimethylallyl diphosphate (DMAPP), a product of both of these pathways, provides a precursor for cytokinin synthesis [53]. Tyagi et al. [54] demonstrated in grapes that cytokinin increased the abundance of phenylalanine ammonia-lyase (PAL) and cinnamate 4-hydroxylase (C4H); our results show that [CO_2_] enrichment increased the abundance of PAL and C4H in cucumber roots under moderate drought stress, possibly promoting the synthesis of flavonoids, total phenols (Figure 6), or lignin. This further confirms our previous findings that cucumber roots under [CO_2_] enrichment have higher free radical scavenging capacity [7].

Meanwhile, after [CO_2_] enrichment, disease resistance protein (RAR1), yellow-leaf-specific gene 9 (YLS9)-like protein, CBS domain-containing protein CBSX3, ferredoxin, and five kinds of major latex protein (MLP)-like proteins all showed differential accumulation in cucumber roots under moderate drought stress (Table S7). Among them, RAR1 can prevent cell dehydration, MLP-like protein can be a positive regulator of downstream signal transduction in response to drought stress [55], YLS9-like protein acts as a late embryogenesis abundant protein to prevent cell dehydration, and CBSX3 and ferredoxin can help maintain dynamic intracellular redox homeostasis by interacting to regulate the level of H_2_O_2_ [56].

[CO_2_] enrichment increased the abundance of xyloglucan endotransglucosylase/hydrolase (XTH), tubulin-folding cofactor (TBC), actin-depolymerizing factor (ADF), and profilin in roots under moderate drought stress (Table S7). Among these, the role of XTH in drought resistance has been confirmed by Cho et al. [57], tubulin and actin are important components of the plant cytoskeleton [58], TBC controls the availability of tubulin subunits and microtubule stability, and profilin and ADF regulate actin as binding proteins. These results indicate that [CO_2_] enrichment can have a positive effect on the dynamic reorganization of the cytoskeleton in cucumber roots under drought stress, especially moderate drought stress [59,60]. In addition, four proteins involved in fatty acid metabolism (FAB2 (acyl-(acyl-carrier-protein) desaturase), accB (acetyl-CoA carboxylase biotin carboxyl carrier protein), DGAT1 (diacylglycerol O-acyltransferase 1), and patellin-6) were upregulated under moderate drought stress, whereas only accB was upregulated under severe drought stress (Table S7). We speculate that [CO_2_] enrichment can maintain the cell membrane integrity of cucumber seedling roots under different degrees of drought stress by increasing the content of unsaturated fatty acids in membrane lipids [61,62].

## 4. Materials and Methods

### 4.1. Plant Material, Growth Conditions, and Experimental Design

**Figure 8:**
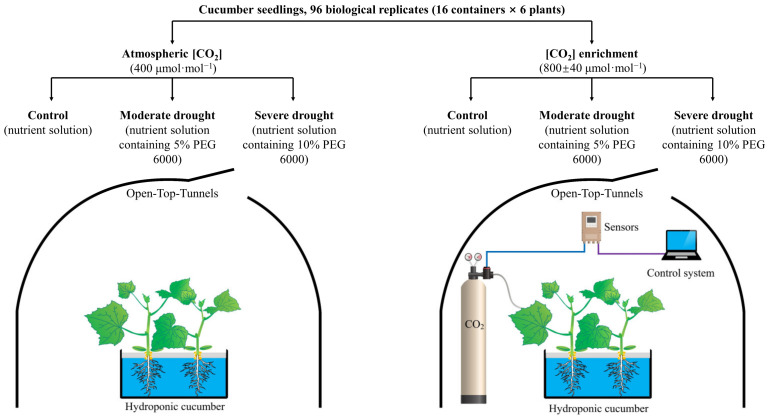
Schematic diagram of experimental design. Cucumber seedlings were placed in four open-top tunnels for hydroponics and two of them were treated with [CO_2_] enrichment using gas cylinders. There were six treatments in total, with 96 biological replicates per treatment.

Cucumber (*Cucumis sativus* L., Cucurbitaceae, ‘Jinyou No. 35’, Tianjin Kernel Cucumber Research Institute, Tianjin, China) was used as test material; uniformly germinated seeds were selected and sown in 50-hole black plastic trays (54 cm length, 28 cm width, and 5 cm height) containing a mixed substrate of peat, perlite, and vermiculite (volume ratio was 3:1:1) and placed in a tunnel for cultivation. After the emergence of the second true leaves of cucumber, uniformly grown seedlings were transplanted into lightproof containers (35 cm length, 28 cm width, and 12 cm height) containing 7 L of Japan Yamazaki nutrient solution (0.5 mM NH_4_H_2_PO_4_, 2.0 mM Ca(NO_3_)_2_·4H_2_O, 3.2 mM KNO_3_, 1.0 mM MgSO_4_·7H_2_O, and full-strength trace elements). Six seedlings were planted in each container, and 16 containers per treatment were used as replicates. There were 96 biological replicates per treatment (16 containers × 6 plants). A split plot design was used; the main plots were [CO_2_] (atmospheric [CO_2_] (A, 400 μmol·mol^−1^) and [CO_2_] enrichment (E, 800 ± 40 μmol·mol^−1^). Liquid CO_2_ cylinders were used to provide CO_2_, infrared absorption principle-based sensors (Auto, Beijing, China) were used to determine [CO_2_], and the split plot factor was drought stress, with PEG 6000 used to simulate drought stress conditions, including control condition (C, nutrient solution), moderate drought stress condition (M, nutrient solution containing 5% PEG 6000, ψ_w_ = −0.05 Mpa), and severe drought stress condition (S, nutrient solution containing 10% PEG 6000, ψ_w_ = −0.15 MPa). Cucumber seedlings were randomly placed in four self-designed open-top tunnels (6 m length, 6 m width, and 2.6 m ridge height), with other environmental factors maintained as described in detail in a previous study (see Figure 8) [8]. The roots of seedlings were sampled on the fifth day of treatment.

### 4.2. Measurements of Biochemical Indices

For each treatment, 15 cucumber plants were randomly selected, then the cucumber root samples were dried to constant weight at 80 °C, mixed, and ground. Soluble sugar and starch contents were determined using 0.5 g samples according to the method of Rosa et al. [63]. Carbohydrates were extracted using 0.1 g samples with 10 mL of 80% (*v*/*v*) ethanol. Sucrose, fructose, and glucose contents were analyzed according to the method in [33]. Total nitrogen content was determined by the Kjeldahl method using 0.2 g of sample digested in a mixture of H_2_SO_4_-H_2_O_2_ [64].

For each treatment, 15 cucumber plants were randomly selected, then the cucumber root samples were mixed and weighed 0.5 g/0.2 g and immediately frozen in liquid nitrogen and stored at −80 °C. Stachyose and raffinose contents were determined by high-performance liquid chromatography (HPLC) according to the method of Lü et al. [65]. Free amino acid content was determined by the ninhydrin reaction [66], and NO_3_^−^-N and NH_4_^+^-N contents were determined by the salicylic acid method [67] and phenol-hypochlorite method [68], respectively. Hexokinase (HK) and MDH were extracted and determined according to the method of Li et al. [33]. Nitrate reductase (NR) activity was determined by the sulfanilic acid method [69]. Glutamate synthase (GOGAT) activity was determined by measuring the decrease in absorption at 340 nm caused by enzymatic oxidation of NADH [33]. Pyruvic acid, citric acid, total phenol and flavonoid contents, and alcohol dehydrogenase (ADH) and glutamate dehydrogenase (GDH) activities were determined using kits according to the manufacturer’s instructions (Comin Biotechnology Co., Ltd., Suzhou, China). Among them, pyruvic acid content was determined using the 2,4-dinitrophenylhydrazine colorimetric method, total phenol content was determined using the Folin-Ciocalteu colorimetric method, citric acid content was determined using the oxidized brominated complex colorimetric method, and flavonoid content was determined using the Al^3+^ colorimetric method. ADH and GDH activities were determined using the decrease in absorption at 340 nm caused by consumption of NADH due to catalytic acetaldehyde and catalytic NH_4_^+^, respectively.

### 4.3. Protein Extraction, Trypsin Digestion, and TMT Labeling

Root samples with 0.2 g were fully ground into powder in liquid nitrogen with a pre-cooled mortar. The powder was combined with a four-fold volume of lysis buffer (containing 10 mM dithiothreitol, 1% protease inhibitor, and 2 mM EDTA) and sonicated. An equal volume of Tris-equilibrated phenol was added prior to centrifugation at 5500× *g* for 10 min at 4 °C. The supernatant was aspirated and added a five-fold volume of 0.1 M ammonium acetate/methanol, then the solution was allowed to form a precipitate overnight. The protein precipitate was washed with methanol and acetone, the precipitate was redissolved with 8 M urea, and the protein concentration was determined with a BCA kit according to the manufacturer’s instructions.

For digestion, the protein solution was reduced with 5 mM dithiothreitol for 30 min at 56 °C and alkylated with 11 mM iodoacetamide for 15 min at room temperature in darkness. The urea concentration of the samples was diluted to below 2 M by adding 0.1 M TEAB. Trypsin was added at a ratio of trypsin to protein of 1:50 (m/m) for the first digestion overnight and at a ratio of trypsin to protein of 1:100 (m/m) for the second 4 h-digestion.

After that, the peptides were desalted using a Strata X C18 SPE column (Phenomenex, Torrance, CA, USA) and then vacuum-dried. The peptides were dissolved in 0.5 M TEAB and labeled according to the TMT kit instructions. Briefly, one unit of TMT reagent was thawed and dissolved in acetonitrile, mixed with the peptides, and incubated for 2 h at room temperature. The resulting labeled peptides were pooled, desalted, and freeze-dried under vacuum. The labeled peptides were first separated into 60 fractions by HPLC using an Agilent 300 Extend C18 column (5 μm particles, 4.6 mm I.D., 250 mm length) with a gradient of 8% to 32% acetonitrile (pH 9.0) over 60 min, then combined into 18 fractions.

### 4.4. LC-MS/MS Analysis, Database Search, and Functional Classification

The combined peptides were dried through vacuum centrifugation and re-dissolved in 0.1% formic acid and 0.2% acetonitrile, then subjected to gradient elution through a homemade reversed-phase analytical column on an EASY-nLC 1200 UPLC system (Thermo, Waltham, MA, USA) with the following settings: 400 nL/min constant flow, start from 8% to 16% solvent (0.1% formic acid in 90% acetonitrile) for over 30 min, 16% to 30% for 15 min, 30% to 80% for 2 min, and hold at 80% for the last 3 min. The peptides were then subjected to an NSI source and tandem mass spectrometry (MS/MS) was performed on a Q Exactive^TM^ Plus hybrid quadrupole-Orbitrap mass spectrometer (Thermo, Waltham, MA, USA) coupled online to UPLC. Briefly, intact peptides were detected in the Orbitrap at a resolution of 60,000 with an MS range of 350–1550 *m*/*z* for full scan. The twenty most intense precursor ions per survey scan were selected for higher-energy collisional dissociation (HCD) fragmentation at a normalized collision energy of 32%, and the resulting fragments were analyzed with the Orbitrap at a resolution of 15,000 with a fixed first mass of 100 *m*/*z*. The mass spectrometer was operated in data-dependent acquisition mode to alternate between one MS scan and twenty MS/MS scans, with dynamic exclusion of 30 s, automatic gain control of 5E4, a maximum inject time of 70 ms, and a signal threshold of 10,000 ions/s.

The resulting MS/MS data were processed using the Maxquant search engine (v. 1.5.2.8). Tandem mass spectra were searched against a proteome database (Uniprot_Cucumis_sativus_3659_PR_20181112.fasta, 23,744 sequences) concatenated with a reverse decoy database. Trypsin/P was specified as the cleavage enzyme, allowing up to two missing cleavages. The mass tolerance for precursor ions was set as 20 ppm in the first search and 5 ppm in the main search, and the mass tolerance for fragment ions was set as 0.02 Da. Carbamidomethyl on Cys was specified as the fixed modification and oxidation on Met was specified as the variable modification. FDR was adjusted to <1%, and the minimum score for peptides was set to >40.

The annotation proteome for Gene Ontology (GO) was derived from the UniProt-GOA database (http://www.ebi.ac.uk/GOA/ accessed on 25 November 2018). Identified proteins domains’ functional descriptions were annotated by InterProScan based on the protein sequence alignment method using the InterPro (http://www.ebi.ac.uk/interpro/ accessed on 25 November 2018) domain database. The Kyoto Encyclopedia of Genes and Genomes (KEGG) database (http://www.genome.jp/kaas-bin/kaas_main accessed on 25 November 2018, http://www.kegg.jp/kegg/mapper.html accessed on 25 November 2018) was used to annotate protein pathways, and Wolfpsort (http://www.genscript.com/psort/wolf_psort.html accessed on 25 November 2018), a subcellular localization predication software, was used to predict subcellular localization. For further hierarchical clustering based on different protein functional classifications (such as GO, Domain, Pathway, and Complex), we first collated all the categories obtained after enrichment along with their *p*-values, then filtered for those categories which were enriched in at least one of the clusters with *p*-value < 0.05. This filtered *p*-value matrix was transformed by the function x = −log10 (*p*-value). Finally, these x values were z-transformed for each functional category. These z scores were then clustered by one-way hierarchical clustering (Euclidean distance, average linkage clustering) in Genesis. Cluster membership was visualized as a heat map using the “heatmap.2” function from the “gplots” R-package.

### 4.5. Statistical Data Analysis

All physiological data were expressed as the mean ± standard deviation (SD) of biological replicates and subjected to analysis of variance (ANOVA) and correlation analysis by SPSS 20 (IBM, New York, NY, USA). When analysis generated a significant *p*-value (*p* < 0.05) for the treatments, the means were compared by Duncan’s new multiple range test. SigmaPlot 12.5 (IBM, New York, NY, USA) was applied to draw graphs.

## 5. Conclusions

**Figure 9:**
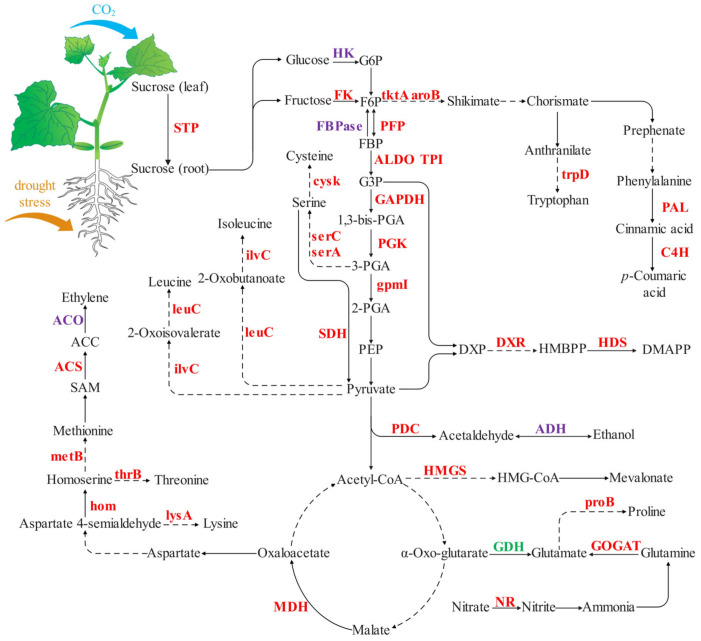
The main differentially accumulating proteins (DAPs) of cucumber seedling root response to [CO_2_] enrichment under drought stress. Black words indicate metabolites, arrows indicate metabolic processes, and omitted processes are indicated by dashed lines. Red words indicate the upregulated DAPs under moderate drought stress after [CO_2_] enrichment, green words indicate the downregulated DAPs under moderate drought stress after [CO_2_] enrichment, and purple words indicate the upregulated DAPs under both moderate and severe drought stress after [CO_2_] enrichment. Abbreviations: STP: sugar transport protein; HK: hexokinase; G6P: glucose 6-phosphate; F6P: fructose 6-phosphate; FK: fructokinase; PFP: pyrophosphate-fructose 6-phosphate 1-phosphotransferase; FBPase: fructose-1,6-bisphosphatase; FBP: fructose 1,6-bisphosphate; ALDO: fructose-bisphosphate aldolase; TPI: triosephosphate isomerase; G3P: glyceraldehyde 3-phosphate; GAPDH: glyceraldehyde-3-phosphate dehydrogenase; 1,3-bis-PGA: 1,3-bisphospho-D-glycerate; PGK: phosphoglycerate kinase; 3-PGA: 3-phosphoglycerate; gpmI: 2,3-bisphosphoglycerate-independent phosphoglycerate mutase; 2-PGA: 2-phospho-D-glycerate; PEP: phosphoenolpyruvate; PDC: pyruvate decarboxylase; ADH: alcohol dehydrogenase; MDH: malate dehydrogenase; tktA: transketolase; aroB: 3-dehydroquinate synthase; trpD: anthranilate phosphoribosyltransferase; PAL: phenylalanine ammonia-lyase; C4H: cinnamate 4-hydroxylase; serA: D-3-phosphoglycerate dehydrogenase; serC: phosphoserine aminotransferase; cysk: cysteine synthase; SDH: serine dehydratase; ilvC: ketol-acid reductoisomerase; leuC: 3-isopropylmalate dehydratase large subunit; lysA: diaminopimelate decarboxylase; hom: homoserine dehydrogenase; thrB: homoserine kinase; metB: cystathionine gamma-synthase; SAM: S-adenosyl-L-methionine; ACC: 1-aminocyclopropane-1-carboxylate; ACS: ACC synthase; ACO: ACC oxidase; NR: nitrate reductase; GOGAT: glutamate synthase; GDH: glutamate dehydrogenase; proB: glutamate 5-kinase/delta-1-pyrroline-5-carboxylate synthase; HMG-CoA: 3-hydroxy-3-methylglutaryl-CoA; HMGS: HMG-CoA synthase; DMAPP: dimethylallyl diphosphate; DXP: 1-deoxy-D-xylulose 5-phosphate; DXR: DXP reductoisomerase; HMBPP: 4-hydroxy-3-methylbut-2-enyldiphosphate; HDS: HMBPP synthase.

This study combined proteomic results supported with additional biochemical data to analyze the major KEGG pathways of DAPs in response to [CO_2_] enrichment and drought stress. [CO_2_] enrichment increased proteins associated with carbohydrate synthesis, energy, and amino acid metabolism in cucumber seedling roots under drought stress (especially moderate drought stress) (Figure 9), significantly induced the expression of proteins involved in stress and defense, cell wall and cytoskeleton metabolic, and effectively maintained the balance of protein processing and degradation, which finally improved the drought resistance of cucumber seedling roots.

## Data Availability

The original contributions presented in the study are publicly available. The mass spectrometry proteomics data have been deposited to the ProteomeXchange Consortium (http://proteomecentral.proteomexchange.org accessed on 26 January 2022) via the iProX partner repository [70] with the dataset identifier PXD031284.

## Supplementary Materials

The supporting information can be downloaded at: https://www.mdpi.com/article/10.3390/ijms232314911/s1.

## References

[1] Zargar S.M., Nagar P., Deshmukh R., Nazir M., Wani A.A., Masoodi K.Z. (2017). Aquaporins as potential drought tolerance inducing proteins: Towards instigating stress tolerance. J. Proteom..

[2] Feng Q., Ma H., Jiang X.M., Wang X., Cao S. (2015). What has caused desertification in China?. Sci. Rep..

[3] Van der Kooi C.J., Reich M., Low M., De Kok L.J., Tausz M. (2016). Growth and yield stimulation under elevated CO_2_ and drought: A meta-analysis on crops. Environ. Exp. Bot..

[4] Ainsworth E.A., Long S.P. (2005). What have we learned from 15 years of free-air [CO_2_] enrichment FACE? A meta-analytic review of the responses of photosynthesis, canopy properties and plant production to rising CO_2_. New. Phytol..

[5] Arndal M.F., Tolver A., Larsen K.S., Beier C., Schmidt I.K. (2018). Fine root growth and vertical distribution in response to elevated CO_2_, warming and drought in a mixed heathland–grassland. Ecosystems.

[6] Pacholski A., Manderscheid R., Weigel H.J. (2015). Effects of free air [CO_2_] enrichment on root growth of barley, sugar beet and wheat grown in a rotation under different nitrogen supply. Eur. J. Agron..

[7] Li Y.M., He X.R., Li Q.M., Liu B.B., Li S.H., Ai X.Z. (2018). Effect of [CO_2_] enrichment on antioxidant system in cucumber seedling root system under drought stress in Chinese with English abstract. Plant Physiol. Commun..

[8] Li Y.M., Li S.H., He X.R., Jiang W.L., Zhang D.L., Liu B.B. (2020). [CO_2_] enrichment enhanced drought resistance by regulating growth, hydraulic conductivity and phytohormone contents in the root of cucumber seedlings. Plant Physiol. Biochem..

[9] Mohammadi P.P., Moieni A., Hiraga S., Komatsu S. (2011). Organ-specific proteomic analysis of drought-stressed soybean seedlings. J. Proteom..

[10] Mohammadi P.P., Moieni A., Komatsu S. (2012). Comparative proteome analysis of drought-sensitive and drought-tolerant rapeseed roots and their hybrid F1 line under drought stress. Amino Acids.

[11] Ghaffari M., Toorchi M., Valizadeh M., Komatsu S. (2013). Differential response of root proteome to drought stress in drought sensitive and tolerant sunflower inbred lines. Funct. Plant Biol..

[12] Lu Y.J., Li N.Y., Sun J., Hou P.C., Jing X.S., Zhu H.P. (2013). Exogenous hydrogen peroxide, nitric oxide and calcium mediate root ion fluxes in two non-secretor mangrove species subjected to NaCl stress. Tree Physiol..

[13] Kaur V., Mahla R., Behl R. (2014). Research Article High temperature, drought and their interaction induced protein alterations in sensitive and tolerant wheat varieties. Electron. J. Plant Breed..

[14] Saibil H. (2013). Chaperone machines for protein folding, unfolding and disaggregation. Nat. Rev. Mol. Cell Biol..

[15] Webb K.M., Broccardo C.J., Prenni J.E., Wintermantel W.M. (2014). Proteomic profiling of sugar beet Beta vulgaris leaves during rhizomania compatible interactions. Proteomes.

[16] Li C.X., Bian B.T., Gong T.Y., Liao W.B. (2018). Comparative proteomic analysis of key proteins during abscisic acid-hydrogen peroxide-induced adventitious rooting in cucumber (*Cucumis sativus* L.) under drought stress. J. Plant Physiol..

[17] Burgess P., Huang B.R. (2014). Root protein metabolism in association with improved root growth and drought tolerance by elevated carbon dioxide in creeping bentgrass. Field Crops Res..

[18] Wang X.L., Cai X.F., Xu C.X., Wang Q.H., Dai S.J. (2016). Drought-responsive mechanisms in plant leaves revealed by proteomics. Int. J. Mol. Sci..

[19] He J.Q., Hu W., Li Y.X., Zhu H.H., Zou J., Wang Y.H. (2022). Prolonged drought affects the interaction of carbon and nitrogen metabolism in root and shoot of cotton. Environ. Exp. Bot..

[20] Gillespie K.M., Xu F.X., Richter K.T., McGrath J.M., Markelz R.J.C., Ort D.R. (2012). Greater antioxidant and respiratory metabolism in field-grown soybean exposed to elevated O_3_ under both ambient and elevated CO_2_. Plant Cell Environ..

[21] Klein T., Hoch G., Yakir D., Korner C. (2014). Drought stress, growth and nonstructural carbohydrate dynamics of pine trees in a semi-arid forest. Tree Physiol..

[22] O’Brien M.J., Leuzinger S., Philipson C.D., Tay J., Hector A. (2014). Drought survival of tropical tree seedlings enhanced by non-structural carbohydrate levels. Nat. Clim. Change.

[23] Aluko O.O., Li C., Wang Q., Liu H. (2021). Sucrose utilization for improved crop yields: A review article. Int. J. Mol. Sci..

[24] Zhou Q., Wang Y., Zhao X., Han L.N., Yang S.J. (2021). Effects of CO_2_ on transplantation of grape plantlets cultured in vitro by promoting photosynthesis. Sci. Hortic..

[25] Calvo O.C., Franzaring J., Schmid I., Fangmeier A. (2019). Root exudation of carbohydrates and cations from barley in response to drought and elevated CO_2_. Plant Soil.

[26] Li X., Dong J., Chu W., Chen Y., Duan Z. (2018). The relationship between root exudation properties and root morphological traits of cucumber grown under different nitrogen supplies and atmospheric CO_2_ concentrations. Plant Soil.

[27] Du J., Guo S.R., Sun J., Shu S. (2018). Proteomic and physiological analyses reveal the role of exogenous spermidine on cucumber roots in response to Ca(NO_3_)_2_ stress. Plant Mol. Biol..

[28] Guo X.M., Ronhovde K., Yuan L.L., Yao B., Soundararajan M.P., Elthon T. (2012). Pyrophosphate-dependent fructose-6-phosphate 1-phosphotransferase induction and attenuation of Hsp gene expression during endosperm modification in quality protein maize. Plant Physiol..

[29] Degenkolbe T., Do P.T., Kopka J., Zuther E., Hincha D.K., Kohl K.I. (2013). Identification of drought tolerance markers in a diverse population of rice cultivars by expression and metabolite profiling. PLoS ONE.

[30] Kappachery S., Baniekal-Hiremath G., Yu J.W., Park S.W. (2015). Effect of over-and under-expression of glyceraldehyde 3-phosphate dehydrogenase on tolerance of plants to water-deficit stress. Plant Cell Tissue Organ Cult..

[31] Zhang X.H., Rao X.L., Shi H.T., Li R.J., Lu Y.T. (2011). Overexpression of a cytosolic glyceraldehyde-3-phosphate dehydrogenase gene OsGAPC3 confers salt tolerance in rice. Plant Cell Tissue Organ Cult..

[32] Diab A., Kantety R., Ozturk Gokce N., Benscher D., Nachit M., Sorrells M. (2008). Drought—Inducible genes and differentially expressed sequence tags associated with components of drought tolerance in durum wheat. Sci. Res. Essays.

[33] Li S.H., Li Y.M., Gao Y., He X.R., Zhang D.L., Liu B.B. (2020). Effects of [CO_2_] enrichment on non-structural carbohydrate metabolism in leaves of cucumber seedlings under salt stress. Sci. Hortic..

[34] Manaa A., Ben Ahmed H., Valot B., Bouchet J.P., Aschi-Smiti S., Causse M. (2011). Salt and genotype impact on plant physiology and root proteome variations in tomato. J. Exp. Bot..

[35] Zadraznik T., Hollung K., Egge-Jacobsen W., Meglic V., Sustar-Vozlic J. (2013). Differential proteomic analysis of drought stress response in leaves of common bean *Phaseolus vulgaris* L.. J. Proteom..

[36] Koh J., Chen G., Yoo M.J., Zhu N., Dufresne D., Erickson J.E. (2015). Comparative proteomic analysis of brassica napus in response to drought stress. J. Proteome Res..

[37] Li S.H., Li Y.M., He X.R., Li Q.M., Liu B.B., Ai X.Z. (2019). Response of water balance and nitrogen assimilation in cucumber seedlings to [CO_2_] enrichment and salt stress. Plant Physiol. Biochem..

[38] Zeng W.J., Peng Y.L., Zhao X.Q., Wu B.Y., Chen F.Q., Ren B. (2019). Comparative proteomics analysis of the seedling root response of drought-sensitive and drought-tolerant maize varieties to drought stress. Int. J. Mol. Sci..

[39] Li X.J., Yang M.F., Chen H., Qu L.Q., Chen F., Shen S.H. (2010). Abscisic acid pretreatment enhances salt tolerance of rice seedlings: *Proteomic evidence*. Biochim. Biophys. Acta (BBA) Proteins Proteom..

[40] Prinsi B., Espen L. (2015). Mineral nitrogen sources differently affect root glutamine synthetase isoforms and amino acid balance among organs in maize. BMC Plant Biol..

[41] Raveneau M.P., Benamar A., Macherel D. (2017). Water content, adenylate kinase, and mitochondria drive adenylate balance in dehydrating and imbibing seeds. J. Exp. Bot..

[42] Bannenberg G., Martínez M., Hamberg M., Castresana C. (2008). Diversity of the enzymatic activity in the lipoxygenase gene family of arabidopsis thaliana. Lipids.

[43] Cui Q.Q., Li Y.M., He X.R., Li S.H., Zhong X., Liu B.B. (2019). Physiological and iTRAQ based proteomics analyses reveal the mechanism of elevated CO_2_ concentration alleviating drought stress in cucumber *Cucumis sativus* L. seedlings. Plant Physiol. Biochem..

[44] Jedmowski C., Ashoub A., Beckhaus T., Berberich T., Karas M., Brüggemann W. (2014). Comparative analysis of sorghum bicolor proteome in response to drought stress and following recovery. Int. J. Proteom..

[45] Kim S.H., Kim S.H., Palaniyandi S.A., Yang S.H., Suh J.W. (2015). Expression of potato *S-adenosyl-L-methionine* synthase SbSAMS gene altered developmental characteristics and stress responses in transgenic *Arabidopsis* plants. Plant Physiol. Biochem..

[46] Wan L.Y., Zhang J.F., Zhang H.W., Zhang Z.J., Quan R.D., Zhou S.R. (2011). Transcriptional activation of OsDERF1 in OsERF3 and OsAP2-39 negatively modulates ethylene synthesis and drought tolerance in rice. PLoS ONE.

[47] Gupta S., Bharalee R., Bhorali P., Bandyopadhyay T., Gohain B., Agarwal N. (2012). Identification of drought tolerant progenies in tea by gene expression analysis. Funct. Integr. Genom..

[48] Lyzenga W.J., Stone S.L. (2012). Abiotic stress tolerance mediated by protein ubiquitination. J. Exp. Bot..

[49] Wan X., Mo A., Liu S., Yang L., Li L. (2010). Constitutive expression of a peanut ubiquitin-conjugating enzyme gene in Arabidopsis confers improved water-stress tolerance through regulation of stress-responsive gene expression. J. Biosci. Bioeng..

[50] Piasecka A., Sawikowska A., Kuczynska A., Ogrodowicz P., Mikolajczak K., Krystkowiak K. (2017). Drought-related secondary metabolites of barley *Hordeum vulgare* L. leaves and their metabolomic quantitative trait loci. Plant J..

[51] Li M., Li Y.M., Zhang W.D., Li S.H., Gao Y., Ai X.Z. (2018). Metabolomics analysis reveals that elevated atmospheric CO_2_ alleviates drought stress in cucumber seedling leaves. Anal. Biochem..

[52] Kiba T., Takebayashi Y., Kojima M., Sakakibara H. (2019). Sugar-induced de novo cytokinin biosynthesis contributes to *Arabidopsis* growth under elevated CO_2_. Sci. Rep..

[53] Vranová E., Coman D., Gruissem W. (2013). Network analysis of the MVA and MEP pathways for isoprenoid synthesis. Annu. Rev. Plant Biol..

[54] Tyagi K., Maoz I., Kochanek B., Sela N., Lerno L., Ebeler S.E. (2021). Cytokinin but not gibberellin application had major impact on the phenylpropanoid pathway in grape. Hortic. Res..

[55] Ma Q.L., Kang J.M., Long R.C., Zhang T.J., Xiong J.B., Zhang K. (2017). Comparative proteomic analysis of alfalfa revealed new salt and drought stress-related factors involved in seed germination. Mol. Biol. Rep..

[56] Costa M.C.D., Righetti K., Nijveen H., Yazdanpanah F., Ligterink W., Buitink J. (2015). A gene co-expression network predicts functional genes controlling the re-establishment of desiccation tolerance in germinated *Arabidopsis thaliana* seeds. Planta.

[57] Cho S.K., Kim J.E., Park J., Eom T.J., Kim W.T. (2006). Constitutive expression of abiotic stress-inducible hot pepper CaXTH3, which encodes a xyloglucan endotransglucosylase/hydrolase homolog, improves drought and salt tolerance in transgenic *Arabidopsis* plants. FEBS Lett..

[58] Jin H.J., Xu M.J., Chen H., Zhang S.R., Han X.Y., Tang Z.H. (2016). Comparative proteomic analysis of differentially expressed proteins in *Amaranthus hybridus* L. roots under cadmium stress. Water Air Soil Pollut..

[59] Takac T., Pechan T., Richter H., Muller J., Eck C., Bohm N. (2011). Proteomics on brefeldin a-treated arabidopsis roots reveals profilin 2 as a new protein involved in the cross-talk between vesicular trafficking and the actin cytoskeleton. J. Proteome Res..

[60] Mondal H.A., Louis J., Archer L., Patel M., Nalam V.J., Sarowar S. (2018). Arabidopsis actin-depolymerizing factor3 is required for controlling aphid feeding from the phloem. Plant Physiol..

[61] Zhong D.H., Du H.M., Wang Z.L., Huang B.R. (2011). Genotypic variation in fatty acid composition and unsaturation levels in bermudagrass associated with leaf dehydration tolerance. J. Am. Soc. Hortic. Sci..

[62] Gasulla F., vom Dorp K., Dombrink I., Zahringer U., Gisch N., Dormann P. (2013). The role of lipid metabolism in the acquisition of desiccation tolerance in *Craterostigma plantagineum*: A comparative approach. Plant J..

[63] Rosa M., Hilal M., González J.A., Prado F.E. (2009). Low-temperature effect on enzyme activities involved in sucrose–starch partitioning in salt-stressed and salt-acclimated cotyledons of quinoa *Chenopodium quinoa* Willd. seedlings. Plant Physiol. Biochem..

[64] Wang D.H., Shi Q.H., Wang X.F., Wei M., Hu J.Y., Liu J. (2010). Influence of cow manure vermicompost on the growth, metabolite contents, and antioxidant activities of Chinese cabbage *Brassica campestris* ssp. chinensis. Biol. Fertil. Soils.

[65] Lü J.G., Sui X.L., Ma S., Li X., Liu H., Zhang Z.X. (2017). Suppression of cucumber stachyose synthase gene CsSTS inhibits phloem loading and reduces low temperature stress tolerance. Plant Mol. Biol..

[66] Aurisano N., Bertani A., Reggiani R. (1995). Involvement of calcium and calmodulin in protein and amino acid metabolism in rice roots under anoxia. Plant Cell Physiol..

[67] Cataldo D.A., Maroon M., Schrader L.E., Youngs V.L. (1975). Rapid colorimetric determination of nitrate in plant tissue by nitration of salicylic acid. Commun. Soil Sci. Plant Anal..

[68] Solorzano L. (1969). Determination of ammonia in natural waters by the phenolhypochlorite method. Limnol. Oceanogr..

[69] Datta R., Sharma R. (1999). Temporal and spatial regulation of nitrate reductase and nitrite reductase in greening maize leaves. Plant Sci..

[70] Ma J., Chen T., Wu S., Yang C., Bai M., Shu K. (2019). iProX: An integrated proteome resource. Nucleic Acids Res..

