# Supplementary material for: Mechanism of [CO2] Enrichment Alleviated Drought Stress in the Roots of Cucumber Seedlings Revealed via Proteomic and Biochemical Analysis
Source: Int J Mol Sci. 2022 Nov 28;23(23):14911. doi: 10.3390/ijms232314911 (PMC9737773; doi:10.3390/ijms232314911)
Supplement: Supplementary file 1 [file ijms-23-14911-s001.zip › ijms-1967589_supp1_to_conv.pdf]

International Journal of *Molecular Sciences*

MDPI

Article: Supplementary materials

Mechanism of [CO_2] Enrichment Alleviated Drought Stress in the Roots of Cucumber Seedlings Revealed via Proteomic and Biochemical Analysis

Yiman Li [1,†], Wendong Zhang [2,†], Dalong Zhang [1,3], Yinjian Zheng [4], Yaliang Xu [4], Binbin Liu [3,*] and Qingming Li [4,*]

[1] College of Horticulture Science and Engineering, Shandong Agricultural University, Tai'an 271018, China
[2] College of Water Resource and Civil Engineering, China Agricultural University, Beijing 100083, China
[3] State Key Laboratory of Crop Biology, Tai'an 271018, China
[4] Institute of Urban Agriculture, Chinese Academy of Agricultural Sciences, Chengdu 610299, China
* Correspondence: klab@sdau.edu.cn (B.L.); liqingming@caas.cn (Q.L.)
† These authors contributed equally to this work.

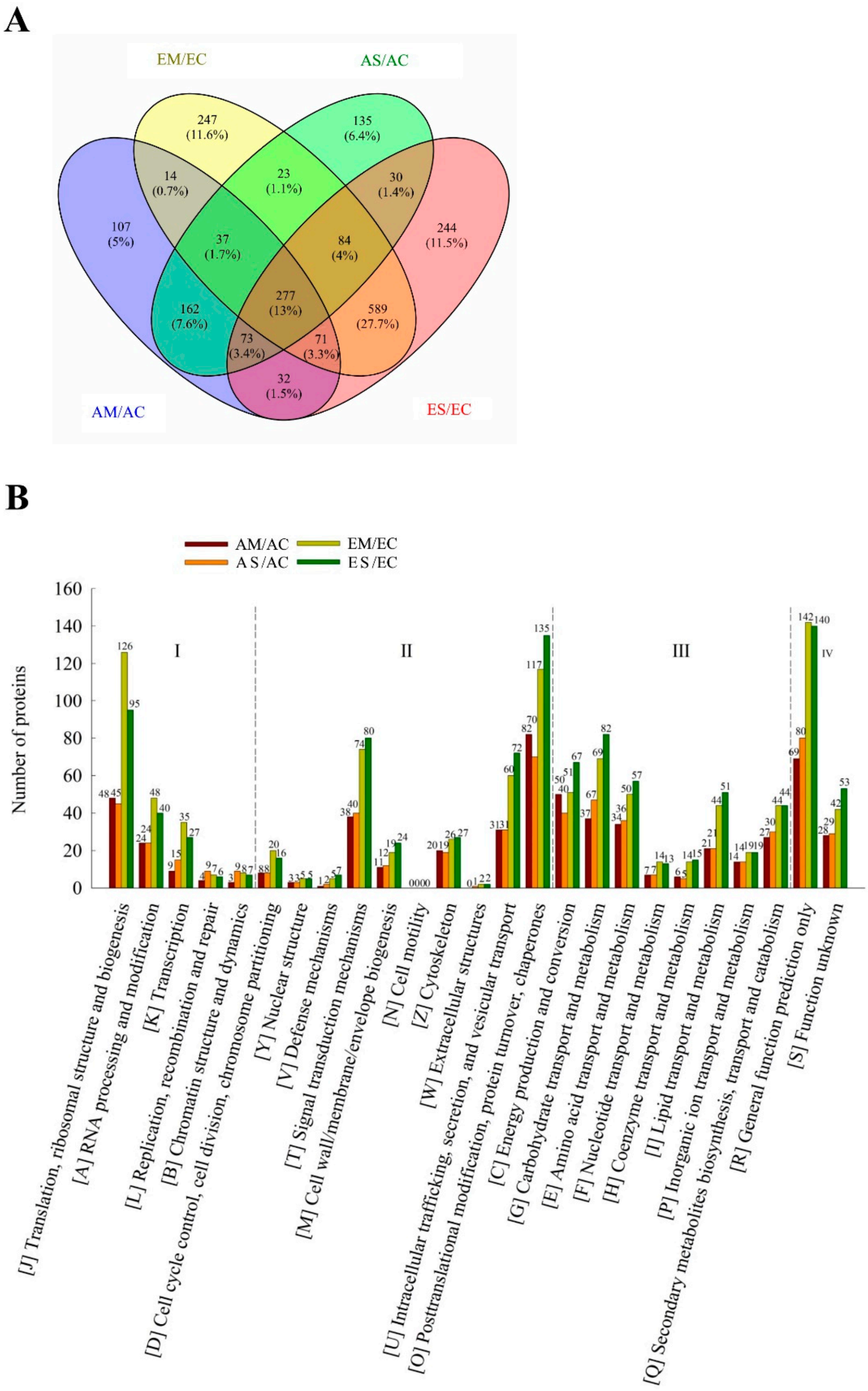

Figure S1. The differential proteins under moderate and severe drought stress. (A) Venn diagram analysis, (B) KOG functional classification chart ((I) Information storage and processing, (II) Cellular processes and signals, (III) Metabolism, (IV) other unknown functions). AC, atmospheric [CO_2] + control condition; EC, [CO_2] enrichment + control condition; AM, atmospheric [CO_2] + moderate drought stress; EM, [CO_2] enrichment + moderate drought stress; AS, atmospheric [CO_2] + severe drought stress; ES, [CO_2] enrichment + severe drought stress. The same below.

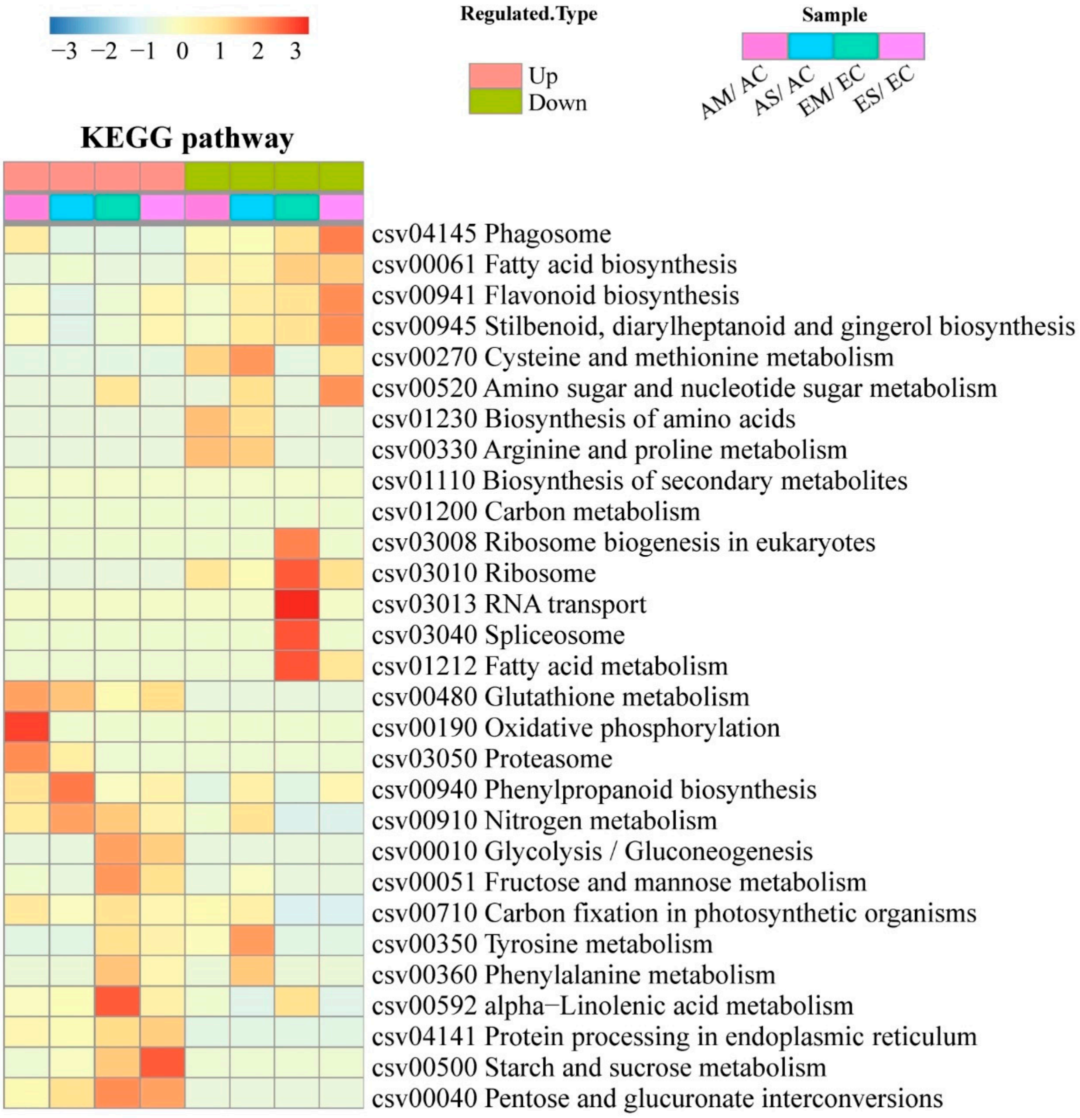

Figure S2. A comprehensive heatmap for cluster analysis of the enrichment patterns of KEGG pathways of differential proteins under moderate and severe drought stress. The color blocks corresponding to the functional description of the differentially expressed proteins in different groups indicate the degree of enrichment, red represents strong enrichment, and blue represents weak enrichment

Table S8. Effects of [CO_2] enrichment on the activities of related enzymes in roots of cucumber seedlings under drought stress.

**Treatments**	**Hexokinase /nmol·min^{-1}·g^{-1}FW**	**Alcohol dehydrogenase /μmol·min^{-1}·g^{-1}FW**	**Malate dehydrogenase /mmol·min^{-1}·g^{-1}FW**	**Nitrate reductase /ugNO_2^-·g^{-1}·h^{-1}FW**	**Glutamate synthase /U·g^{-1}FW**	**Glutamate dehydrogenase /U·g^{-1}FW**
AC	3.78±0.22a	0.10±0.00c	1.35±0.02b	10.16±0.26b	0.13±0.01b	0.661±0.016b
EC	4.01±0.22a	0.13±0.02bc	1.61±0.28a	11.60±1.03a	0.20±0.02a	0.743±0.032a
AM	2.45±0.39b	0.17±0.04b	0.86±0.03c	8.10±0.50c	0.08±0.01cd	0.484±0.033c
EM	2.97±0.34b	0.19±0.07b	1.14±0.11b	9.81±0.20b	0.12±0.01bc	0.335±0.037d
AS	2.45±0.22b	0.19±0.01b	0.53±0.09d	6.97±0.15d	0.03±0.01d	0.343±0.037d
ES	2.67±0.22b	0.32±0.03a	0.71±0.06cd	6.84±0.33d	0.05±0.01d	0.196±0.054e
			p-value			
[CO_2]	0.030*	0.007***	0.002***	0.001***	0.000***	0.001***
Drought	0.000***	0.000***	0.000***	0.000***	0.000***	0.000***
Interaction	0.579^{ns}	0.047*	0.775^{ns}	0.017*	0.009***	0.000***

Note: Different letters within a column indicate a significant difference ($p < 0.05$). AC, atmospheric [CO_2] + control condition; EC, [CO_2] enrichment + control condition; AM, atmospheric [CO_2] + moderate drought stress; EM, [CO_2] enrichment + moderate drought stress; AS, atmospheric [CO_2] + severe drought stress; ES, [CO_2] enrichment + severe drought stress. All results were expressed as the mean ± standard deviation (SD) of three repeated values, *, difference is significant at the 0.05 level; **, difference is significant at the 0.01 level; ***, difference is significant at the 0.001 level. The same as below.

Table S9. Effects of [CO_2] enrichment on the contents of non-structural carbohydrates in roots of cucumber seedlings under drought stress.

**Treatments**	**Starch /mg·g^{-1}DW**	**Total sugar /mg·g^{-1}DW**	**Sucrose /mg·g^{-1}DW**	**Reducing sugar /mg·g^{-1}DW**	**Glucose /mg·g^{-1}DW**	**Fructose /mg·g^{-1}DW**	**Raffinose /mg·g^{-1}FW**	**Stachyose /mg·g^{-1}FW**
AC	280.05±14.97b	138.05±23.94b	21.81±1.99e	65.05±1.25c	1.65±0.09c	8.53±0.56de	1.26±0.04e	1.39±0.03e
EC	324.68±11.89a	143.89±13.62b	24.43±1.54de	69.02±1.24bc	1.93±0.08b	9.06±0.17d	1.33±0.02de	1.42±0.02de
AM	211.14±20.46cd	182.40±22.10ab	26.77±1.85cd	72.80±2.71bc	1.96±0.05b	9.63±0.34cd	1.45±0.03c	1.46±0.02d
EM	246.50±6.96bc	203.44±46.05a	28.46±1.88c	76.43±6.74b	2.13±0.05a	10.55±0.05c	1.42±0.03cd	1.68±0.02a
AS	154.41±21.99e	218.13±29.35a	36.62±2.92b	92.89±5.02a	2.19±0.12a	13.71±0.22b	2.56±0.11b	1.61±0.03b
ES	186.81±33.10de	222.82±32.57a	47.18±2.18a	94.58±6.67a	2.25±0.08a	18.68±1.79a	2.80±0.09a	1.54±0.05c
				p-value				
[CO_2]	0.002***	0.467*	0.000***	0.176^{ns}	0.001***	0.000***	0.008***	0.001***
Drought	0.000***	0.002***	0.000***	0.000***	0.000***	0.000***	0.000***	0.000***
Interaction	0.861^{ns}	0.869^{ns}	0.006***	0.898^{ns}	0.107^{ns}	0.001***	0.010*	0.000***

Table S10. Effects of [CO_2] enrichment on the contents of metabolism related compounds in roots of cucumber seedlings under drought stress.

Treatments	Total nitrogen /%	NH_4^+-N /ug·g^{-1}FW	NO_3^--N /ug·g^{-1}FW	Free amino acids /mg·g^{-1}FW	Pyruvic acid /μg·g^{-1}FW	Citric acid /μmol·g^{-1}FW	Total phenols /mg·g^{-1}DW	Flavonoid /mg·g^{-1}DW
AC	3.15±0.07a	16.76±0.76e	430.56±4.37a	0.050±0.005a	17.08±3.05b	28.54±1.30c	1.99±0.17ab	2.32±0.16b
EC	3.18±0.07a	20.32±0.88d	412.25±25.85a	0.054±0.011a	28.49±3.62a	25.63±1.65c	1.47±0.43c	2.29±0.01b
AM	2.41±0.07c	35.81±1.52a	233.59±33.36bc	0.043±0.001a	10.46±1.24c	46.25±1.08b	1.84±0.14abc	2.52±0.10ab
EM	2.66±0.21b	25.90±1.52c	264.52±17.60b	0.048±0.004a	16.83±2.68b	43.96±3.66b	2.25±0.20a	2.62±0.05a
AS	1.77±0.12d	30.22±1.16b	206.44±11.52c	0.040±0.004a	7.71±1.87c	53.13±1.25a	1.66±0.04bc	1.92±0.17c
ES	1.93±0.11d	29.21±1.59b	259.47±6.83b	0.044±0.001a	9.89±1.99c	52.71±1.57a	1.73±0.12bc	2.04±0.17c
				p-value				
[CO_2]	0.022*	0.002***	0.035*	0.130^{ns}	0.000***	0.065^{ns}	0.885^{ns}	0.314^{ns}
Drought	0.000***	0.000***	0.000***	0.021*	0.000***	0.000***	0.032*	0.000***
Interaction	0.333^{ns}	0.000***	0.023*	0.990^{ns}	0.027*	0.534^{ns}	0.011*	0.535^{ns}
